# Composite Scaffolds for Bone Tissue Regeneration Based on PCL and Mg-Containing Bioactive Glasses

**DOI:** 10.3390/biology10050398

**Published:** 2021-05-04

**Authors:** Mauro Petretta, Alessandro Gambardella, Marco Boi, Matteo Berni, Carola Cavallo, Gregorio Marchiori, Maria Cristina Maltarello, Devis Bellucci, Milena Fini, Nicola Baldini, Brunella Grigolo, Valeria Cannillo

**Affiliations:** 1 IRCCS–Istituto Ortopedico Rizzoli, Laboratory RAMSES, Via di Barbiano 1/10, 40136 Bologna, Italy; mauro.petretta@ior.it (M.P.); carola.cavallo@ior.it (C.C.); brunella.grigolo@ior.it (B.G.); 2 RegenHU LTD, Z.I. Du Vivier 22, CH-1690 Villaz-St-Pierre, Switzerland; 3 IRCCS–Istituto Ortopedico Rizzoli, Surgical Sciences and Technologies Complex Structure, Via di Barbiano 1/10, 40136 Bologna, Italy; alessandro.gambardella@ior.it (A.G.); gregorio.marchiori@ior.it (G.M.); milena.fini@ior.it (M.F.); 4 IRCCS–Istituto Ortopedico Rizzoli, Laboratory for Nanobiotechnology-NaBi, Via di Barbiano 1/10, 40136 Bologna, Italy; nicola.baldini@ior.it; 5 IRCCS–Istituto Ortopedico Rizzoli, Medical Technology Laboratory Complex Structure, Via di Barbiano 1/10, 40136 Bologna, Italy; matteo.berni@ior.it; 6 IRCCS–Istituto Ortopedico Rizzoli, BST Biomedical Science and Technologies Laboratory, Via di Barbiano 1/10, 40136 Bologna, Italy; mariacristina.maltarello@ior.it; 7 Department of Engineering “Enzo Ferrari”, University of Modena and Reggio Emilia, Via P. Vivarelli 10, 41125 Modena, Italy; devis.bellucci@unimore.it (D.B.); valeria.cannillo@unimore.it (V.C.)

**Keywords:** PCL, bioactive glasses, therapeutic ions, magnesium, composite scaffolds, human bone-marrow-derived mesenchymal stem cells, tissue engineering, bone

## Abstract

**Simple Summary:**

Polycaprolactone (PCL) is a bioresorbable and biocompatible polymer that has been widely used in long-term implants. However, when it comes to regenerative medicine, PCL suffers from some shortcomings such as a slow degradation rate, poor mechanical properties, and low cell adhesion. The incorporation of ceramics such as bioactive glasses into the PCL matrix has yielded a class of hybrid biomaterials with remarkably improved mechanical properties, controllable degradation rates, and enhanced bioactivity, which are suitable for bone tissue engineering. The use of conventional approaches (such as solvent casting and particulate leaching, phase separation, electrospinning, freeze drying, etc.) in realizing these composite scaffolds strongly affects the control of both the internal and the external architecture of scaffolds, including pore size, pore morphology, and overall structure porosity. Accordingly, 3D printing was used in this study because of the benefits offered over conventional methods, such as high flexibility in shape and size, high reproducibility, capabilities of precise control over internal architecture down to the microscale level, and a customized design that can be tailored to specific patient needs. The optimization of the scaffold structure was previously investigated in terms of architecture through the combination of the Taguchi method and CAD drawing, and, in this study, it was investigated by varying the composition of the composite material.

**Abstract:**

Polycaprolactone (PCL) is widely used in additive manufacturing for the construction of scaffolds for tissue engineering because of its good bioresorbability, biocompatibility, and processability. Nevertheless, its use is limited by its inadequate mechanical support, slow degradation rate and the lack of bioactivity and ability to induce cell adhesion and, thus, bone tissue regeneration. In this study, we fabricated 3D PCL scaffolds reinforced with a novel Mg-doped bioactive glass (Mg-BG) characterized by good mechanical properties and biological reactivity. An optimization of the printing parameters and scaffold fabrication was performed; furthermore, an extensive microtopography characterization by scanning electron microscopy and atomic force microscopy was carried out. Nano-indentation tests accounted for the mechanical properties of the scaffolds, whereas SBF tests and cytotoxicity tests using human bone-marrow-derived mesenchymal stem cells (BM-MSCs) were performed to evaluate the bioactivity and in vitro viability. Our results showed that a 50/50 wt% of the polymer-to-glass ratio provides scaffolds with a dense and homogeneous distribution of Mg-BG particles at the surface and roughness twice that of pure PCL scaffolds. Compared to pure PCL (hardness H = 35 ± 2 MPa and Young’s elastic modulus E = 0.80 ± 0.05 GPa), the 50/50 wt% formulation showed H = 52 ± 11 MPa and E = 2.0 ± 0.2 GPa, hence, it was close to those of trabecular bone. The high level of biocompatibility, bioactivity, and cell adhesion encourages the use of the composite PCL/Mg-BG scaffolds in promoting cell viability and supporting mechanical loading in the host trabecular bone.

## 1. Introduction

Bone tissue engineering aims to mimic the biological environment. Thus, it aims to drive cells toward a defined differentiation pathway and to obtain newly formed functional tissue as a replacement for injured sites. In this perspective, one of the main challenges concerns the development of 3D, degradable, and porous structures simultaneously capable of bearing mechanical loads [1] and transmitting suitable mechanical stimuli to cells during tissue differentiation [2]. The design and fabrication of such structures must allow an optimal control of their internal and external architecture to achieve desired mass transport properties—i.e., permeability and diffusion—and mechanical functionality; not least, it is desirable to have structures to be customized within arbitrary and complex anatomical shapes [3].

With the latest advancements of 3D scanning, design software, and printing technologies, additive manufacturing of individually customized tissue scaffolds can be created for clinical use [4]. In clinical practice, the design of the scaffold structure starts from patients’ medical images acquired with non-invasive techniques, i.e., magnetic resonance imaging or computerized tomography, which are then imported into a manufacturing software that transforms the data acquired into a precise and patient-specific replication of the architecture of the bone defect, facilitating the surgical placement. Finding the right materials combination to enable targeted functionality in terms of biological and mechanical performances, along with developing and optimizing the corresponding additive manufacturing approach, constitutes a major challenge in 3D printing research for clinical applications.

Polycaprolactone (PCL) is a thermoplastic polymer with a low glassy transition and a low melting temperature [5]; it is biocompatible and bioresorbable and can be easily modelled by means of temperature-dependent processes and, thus, has been widely used in melt-extrusion-based approaches for tissue engineering [6,7,8,9]. Nevertheless, its slow degradation rate—due to its high degree of crystallinity and hydrophobicity [10]—, poor mechanical properties [11], and low cell adhesion [12] constitute several drawbacks that could be overcome by means of the inclusion of compounds specific to certain applications; for example, inorganic bioactive materials such as calcium phosphates (CaPs) and bioactive glasses (BGs) were used as micro-fillers in the PCL matrix in some bone repair applications, obtaining composites with an improved elastic modulus and ultimate strength [13,14]. Immediately after implantation, these materials form a biologically active layer of hydroxyl carbonate apatite similar to the bone mineral phase, providing an excellent interfacial bonding between the scaffold and the bone; moreover, both CaPs and BGs can be tailored to deliver ions, e.g., Si, Mg, and Sr, at concentrations capable of improving differentiation and osteogenesis [15,16]. Mg-containing BGs are often used because they regulate the active calcium transport [17,18].

In a previous study, the authors highlighted the capability of computer-aided design (CAD), design of experiments (DOE), the Taguchi method, and finite element analysis (FEA) to implement and optimize the design of precision extrusion deposition (PED) technology in fabricating scaffolds for trabecular bone tissue engineering [19]. In particular, our previous study took into account the ideal scaffold structure available in the literature, i.e., a porosity of 50%, which obtained the best cellular response [20] and corresponded to the best mechanical performance (a compressive strength of 100 GPa for trabecular bone [21]), allowing the selection of the appropriate architecture within the instrumental limits [22].

In this work, we presented for the first time a comprehensive and multifactorial characterization of a 3D-printed composite made of PCL and a novel Mg-doped BG [15]. Such a promising BG was selected because of its remarkable bioactivity and cytocompatibility [15]. The addition of MgO to the parent glass exerted a strong stimulating effect on cell growth and improved bioactivity [15]. PCL was considered to be the most suitable candidate for the investigation because of the aforementioned favorable properties as well as its compatibility with the PED process. While addition of BG to different polymer matrices, such as poly-lactic acid (PLA), has been successfully exploited to produce scaffolds with good mechanical properties and biological response, and with reduced degradation times compared to PCL, these formulations may present different drawbacks. PLA is known to have biodegradation products that decrease pH in surrounding tissues, which can induce inflammation and an autoimmune response, even if this disadvantage can be addressed by its combination with bioceramics [23,24]. Most importantly, though PLA has been proven to be processable through additive manufacturing techniques, it is certainly a more challenging material to process, because of its relevantly higher melting temperature compared to PCL and its likelihood to undergo thermal degradation phenomena for longer residence times within the extruder tank if ad hoc measures like the use of an inert gas feed are not undertaken. For this reason, most of the studies concerning fabrication of PLA/BG composites through melt-based additive manufacturing technologies rely on fused deposition Modeling approaches, requiring an intermediate step of filament fabrication [25]. The high thermal stability and processability of PCL, on the other hand, enables an easier fabrication of the composite scaffolds through PED, eliminating this intermediate step and thus potentially leading to a reduction in process complexity, fabrication times, and material waste.

The focus of this study was on two main tasks: (i) the optimization of the PED printing parameters to match the morphology and inner architecture according to the guidelines stated formerly [19] and (ii) the optimization of the scaffolds’ composition in terms of the relative proportions of its constituents, with the final aim of achieving together enhanced biological performance and suitable mechanical behaviour. To this aim, an extensive characterization of microstructural and micromechanical properties of the obtained constructs was performed and accompanied by an assessment of in vitro tests of bioactivity by simulated body fluid (SBF) and cell viability by Alamar Blue^®^ and Lactate dehydrogenase (LDH); for the latter, human bone-marrow-derived mesenchymal stem cells (BM-MSC) were employed.

## 2. Materials and Methods

### 2.1. Preparation

#### 2.1.1. Preparation of Glass Powders

The bioactive glass BG-Mg was produced by means of a classical melt-quenching route, as previously reported [15,16]. Briefly, the raw powder reagents (SiO_2_, Ca_3_(PO_4_)_2_, Na_2_CO_3_, CaCO_3_, K_2_CO_3_, and Mg(OH)_2_·5H_2_O, Carlo Erba Reagenti, Rodano-Milano, Italy) were weighted, mixed for two hours in a laboratory shaker, and then melted at 1450 °C in a Pt crucible in air. The molten glass was then rapidly quenched in water (at room temperature) to obtain a frit; the frit was subsequently left to dry at 110 °C for 12 h. Finally, the frit was ground and sieved to obtain a powder with a final grain size below 25 μm. The resulting bioactive glass powder had the following composition in mol%: 47.2% SiO_2_, 2.6% P_2_O_5_, 2.3% Na_2_O, 2.3% K_2_O, 35.6% CaO, and 10% MgO [15].

#### 2.1.2. Poly(ε-Caprolactone)/Bioactive Glass Composite Preparation

PCL/BG-Mg composite pellets were prepared for the fabrication of scaffold fibers by PED. Briefly, PCL pellets (MW = 80,000, Sigma Aldrich, St. Louis, MO, USA) were dissolved in chloroform (Sigma Aldrich) and BG-Mg particles were gradually added to the solution under magnetic stirring at room temperature until the desired amounts of polymer-to-particles weight ratios of 70/30 and 50/50 wt%, respectively, were reached. The final solutions were left stirring overnight to guarantee a proper mixing. To further optimize the particles’ dispersion, the composite solution was successively sonicated for 30 min before precipitation in order to avoid clustering. The solutions were then air-dried for 24 h in a chemical hood to guarantee complete solvent evaporation. Finally, the obtained composites were pelletized to be loaded within the PED printhead tank. In the following, PCL will denote the pure polymeric scaffold, while 70/30 and 50/50 will denote the two composite formulations of PCL/BG-Mg, as specified above.

#### 2.1.3. Precision Extrusion Deposition

Using BioCAD software (REGENHU, Switzerland), 5 × 5 × 3 mm^3^ scaffolds were designed. Consistent with a previous study from our group [19], the architecture of the scaffold was set as follows: a single fibre diameter (FIBRE) of 330 µm (330 µm nozzle diameter) and a fibre–fibre distance (PORE) of 300 µm. For cellular tests, an OFFSET between the planes equal to half the distance between fibre centres was introduced in the scaffold architecture to provide an increased surface for cell attachment post-seeding. The PED process was performed by using a 3D Discovery Printer (RegenHU). Aiming to obtain a homogeneous melt, the pellets were kept at the printing temperature and pressure for 30 min before performing the process. Preliminarily, control PCL scaffolds were fabricated according to a previously optimized procedure by setting the following parameters: T = 105 °C, P = 3 Bar, printing speed = 4 mm/s, and screw rotation speed = 18 rpm. Then, optimal printing conditions were investigated to obtain the 70/30 and 50/50 formulations. The 70/30 formulation was obtained at the T and P indicated above but with a lower printing speed (1 mm/s), while the screw rotation speed varied from 12 to 24 rpm for optimizing the process. The 50/50 formulation was then obtained at unvaried parameters, besides a slight temperature increase (115 °C), which allowed the viscosity of the fluid to be reduced as consequence of the increase in the glass vs. polymer ratio. An optical microscope (Eclipse 90I, Nikon, Tokyo, Japan) was used to check the correspondence between the FIBRE and PORE dimensions and the design parameters before further characterizations. To this aim, ImageJ software (v1.49; National Institutes of Health, Bethesda, MD, USA) was used to extract the mean fibre thickness and pore area from several representative optical images.

### 2.2. Characterization Methods

#### 2.2.1. Scanning Electron Microscopy (SEM) Analysis

The morphological analysis of the scaffolds was carried out by SEM (ESEM Quanta 200, FEI Co., Eindhoven, The Netherlands) in order to check the physical integrity of the composites and the presence of the BG-Mg particles on both the surface and in the inner structure of the fibres. To avoid mechanical stress and consequent macroscopic distortion of the structure, the scaffolds were shortly placed into a bath of liquid nitrogen and then cut by a doctor blade along the sagittal plane. Then, all samples were Au-coated (with a thickness of ~4 nm) by DC sputtering. The SEM used was equipped with an X-ray energy dispersion spectroscopy (EDS) analyzer.

#### 2.2.2. Scanning Probe Analysis

Atomic force microscopy (AFM) was carried out in the tapping mode of operation directly on the surface of the scaffolds. Topographies and phase images were acquired at 512 × 512 pixels resolution in air at room temperature by a stand-alone NT-MDT microscope (NT-MDT Co., Moscow, Russia) equipped with silicon cantilevers with a typical tip curvature radius of 10 nm and a resonant frequency around 240 kHz.

#### 2.2.3. Nanomechanical Testing

Nanoindentation analyses on printed fibres were carried out using an NHT2 nanoindentation tester equipped with a diamond Berkovich tip (CSM Instruments, Anton Paar, Peseux, Switzerland). Before testing, the indenter tip was calibrated on a fused quartz reference sample (certificated plane strain modulus E* = 75.1 ± 0.4 GPa) through a calibration intensive mode (110 indentations using 22 different loads, from 0.1 to 100 mN); such calibration allowed the proper definition of the projected contact area (A_C_) from the contact penetration depth (h_C_). Fibre mechanical behaviour was then evaluated on monolayers printed directly onto microscope slides and not on 5 × 5 × 3 mm^3^ scaffolds in order to avoid compliance phenomena between the scaffold layers. The top of the fibre was selected by an optical microscope (Olympus M Plan N) connected to the indenter head. The Elastic modulus (E) and the hardness (H) of the composite materials were calculated by the Doerner–Nix method [26], which approximates the unloading curve to a power law and assumes a purely elastic material response in the first phase of unloading. In testing polymers and, more generally, viscoelastic materials by nanoindentation, it is common practice to use a trapezoidal indentation profile with a short unloading and an extended creep time to reduce time-dependent phenomena [27]. Therefore, all indentations were realized by following a trapezoidal loading profile with loading and unloading time of 10 s and a pause of 200 s, where the latter was determined by creep analyses (a common method is to maintain the applied force at a constant maximum value and, then, measure the change in H and E as a function of creep, i.e., pause). The maximum load (P_MAX_) was varied in the range of 100–450 mN in order to observe the dependence of E and H with the penetration depth (h_MAX_). In this perspective, the chosen P_MAX_ must produce an indentation contact radius a_C_ large enough—in relation to the average particle size—to provide meaningful information about the mechanical properties of the composite (and not of the individual particles or of the polymer matrix) but also a relative h_MAX_ small enough to avoid the influence of the substrate [28]. In this work, powder was sifted by a sieve with a mesh of 67 μm [15] and compared with a_C_, calculated as [29]:

a_C_ = h_C_·tan θ, (1)

where θ = 70.3° is the half-included angle of the Berkovich tip.

#### 2.2.4. In Vitro Bioactivity

The bioactivity of the composite scaffolds was investigated by soaking them in 25 mL of simulated body fluid (SBF) solution, according to the protocol originally developed by Kokubo and Takadama (2006) [30]. The samples were stored in plastic flasks and maintained at 37 °C; the solution was refreshed every 48 h to simulate the body fluid circulation. Samples were removed from the SBF solution after 1, 3, 7, and 14 days, rinsed in distilled water, and dried at room temperature. SEM and EDS were used to evaluate the formation of a superficial hydroxyapatite layer.

#### 2.2.5. Cell Seeding onto PCL Scaffolds

Human bone-marrow-derived mesenchymal stem cells (BM-MSCs), isolated from the bone marrow of two anonymous human donors, were selected from a batch stored in the biobank of our lab at the Rizzoli Orthopaedic Institute. Cells were thawed, expanded for three passages, and seeded onto the scaffolds (pure PCL, 70/30, and 50/50) at the concentrations of 7.0 × 10^5^ and 3.5 × 10^5^. The BM-MSCs were allowed to adhere to scaffolds for at least 30 min at room temperature (RT) and then α-MEM 15% FBS (Thermofisher Scientific, Waltham, MA, USA) was added. The culture medium was changed twice a week. On days 0, 14, and 21 constructs were analysed to evaluate cell viability and cytotoxicity.

#### 2.2.6. Cell Viability Assay

The viability of the BM-MSCs seeded onto the PCL, 70/30, and 50/50 scaffolds were assessed on days 0, 14, and 21 by the Alamar Blue test (Bio-Rad, Hercules, CA, USA) according to the manufacturer’s instructions. In particular, constructs were incubated with 10% of Alamar Blue solution, and after 4 h the fluorescence was read at the 490ex–540em nm wavelength, using a microplate reader (CytoFluorTM 2350, Millipore, Bedford, MA, USA). The results were expressed as a percentage of Alamar Blue reduction.

#### 2.2.7. Cytotoxic Assay

The cytotoxic assay was evaluated on the BM-MSCs seeded onto the PCL, 70/30, and 50/50 scaffolds on days 0, 14, and 21 by the lactate dehydrogenase (LDH) test (cytotoxicity detection kit (LDH), Roche, Basel, Switzerland). LDH measurement was performed on supernatants, according to the manufacturer’s protocol, to test cytotoxicity. Colorimetric detection of LDH was performed at 492–620 nm by a TECAN instrument and values were calculated with reference to the control (scaffolds only).

Results obtained by the Alamar Blue and the LDH assays were analyzed using the GraphPad Prism 5.0 software (GraphPad Software, La Jolla, CA, USA) and expressed as means and standard deviations (mean ± SD). Comparisons among multiple groups were carried out with the Kruskal–Wallis test, followed by Dunn’s post-hoc test. A value of * *p* < 0.05 was considered significant.

## 3. Results

### 3.1. Optimization of the Scaffold Architecture

**Figure 1:**
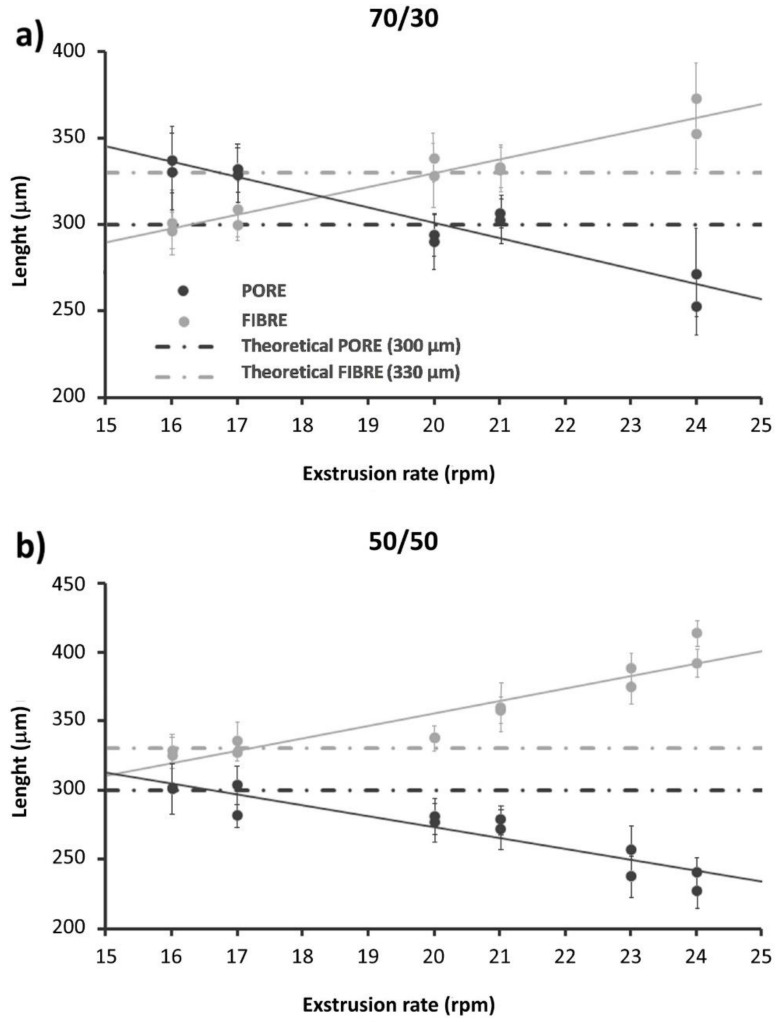
Fibre diameter (FIBRE, light grey) and pore size (PORE, hard grey) as a function of the extrusion rate, evaluated by optical microscope for (**a**) 70/30 and (**b**) 50/50 scaffolds. ImageJ software for dimensional analysis of fibres and pores was used. The results are reported as mean ± SD.

Fidelity of the fabricated structures to design parameters was evaluated for the two formulations in order to identify optimal printing conditions. Clearly all the fabrication parameters (barrel temperature, printing speed, and extrusion rate) influence the thickness of the fibres. Typically, the barrel temperature is set as close as possible to the melting temperature, so as not to degrade it while still guaranteeing flow through the nozzle. Low print speeds were also preferred, since they allow better reproducibility of fibre thicknesses, pore sizes, and orientation between successive fibre planes. For these reasons, the screw rotation speed (indicated as the extrusion rate in Table 1 and Figure 1) was chosen as the main variable parameter in the fidelity optimization process. In both the formulations, as expected, PORE decreased and FIBRE increased by rising the extrusion rate (Figure 1).

Printing parameters to obtain the nominal values of PORE and FIBRE for the 70/30 and 50/50 composites and for pure PCL (the control group) were then collected, as reported in Table 1. Obtained averaged PORE and FIBRE values for the reported sets of parameters were the following: for the 70/30 composites, a PORE value of 292 ± 12 µm and a FIBRE value of 333 ± 18 µm; for the 50/50 composite, a PORE value of 293 ± 14 µm and a FIBRE value of 331 ± 14 µm. Furthermore, a validation of the previously optimized printing parameters for pure PCL was performed, resulting in a PORE value of 297 ± 10 µm and a FIBRE value of 334 ± 14 µm.

### 3.2. SEM Analysis

**Figure 2:**
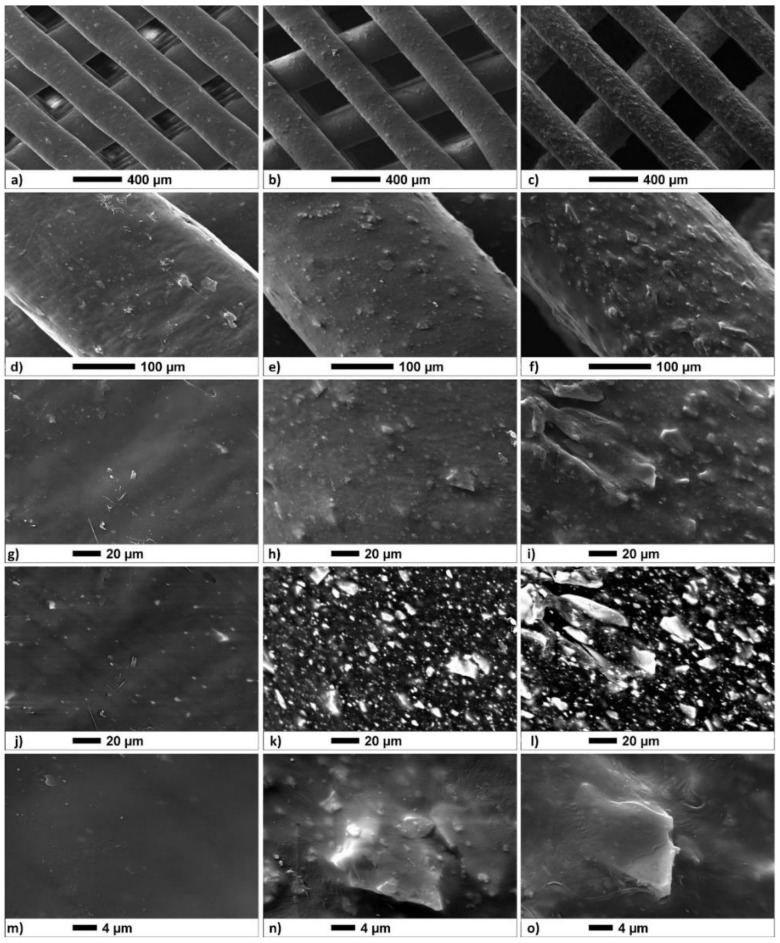
SEM images of top view of the (**a**,**d**,**g**,**j**,**m**) pure PCL, (**b**,**e**,**h**,**k**,**n**) 70/30, and (**c**,**f**,**i**,**l**,**o**) 50/50 compositions. Images were acquired by a secondary electron (SE) detector, except (**j**–**l**), which were achieved through a backscattering electron (BSE) detector, which localized the presence of heavy ions in the samples. Images (**g**–**i**) and (**j**–**l**) were acquired over the same area by the SE and the BSE, respectively.

**Figure 3:**
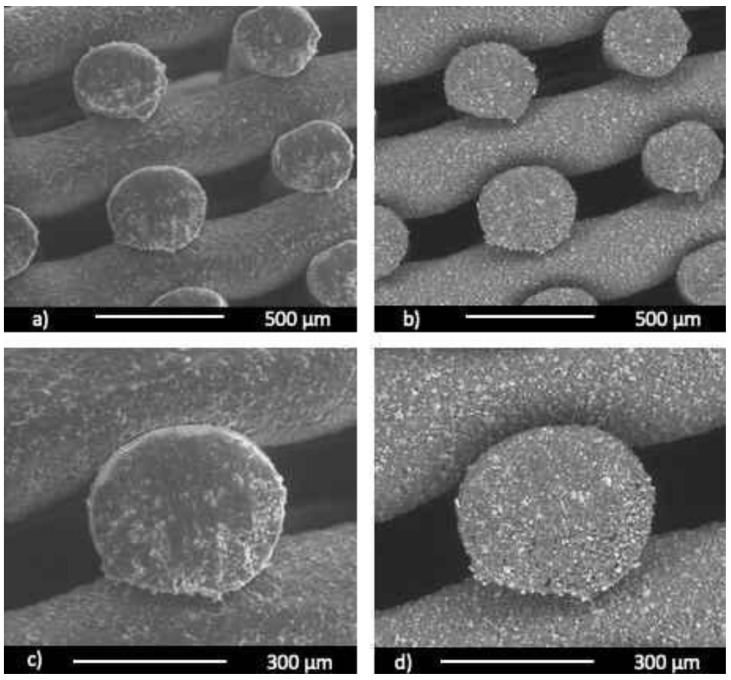
Representative SEM images of a cross-section view of the 50/50 scaffold: (**a**,**c**) secondary electron detector; (**b**,**d**) backscattered electron detector.

SEM imaging corroborated the dimension extrapolated through preliminary optical analysis. Images at low magnification of the top (Figure 2a–c) and side views (Figure 3a,b) of the scaffold clearly showed a well-bounded structure with a uniform PORE distribution and a clear orthogonal orientation between successive fibre planes. The surface roughness of the fibres apparently increased as the BG-Mg concentration increased. However, despite the 50/50 samples showing a greater quantity of particles protruding from the surface compared to the 70/30 samples, no particle detachment was observed in both. Moreover, this evidence was also notable at higher magnifications and considering the backscattered mode acquisition, highlighting both the heavy atoms of BG-Mg (Si, P, and Ca, Figure 2k,l) and the wt%. The BG-Mg particles’ sizes ranged from 1 to 25 μm in agreement with the powder size, which have an irregular shape. These particles appeared uniformly distributed on the surface and in the cross section of the samples (see Figure 3), despite being almost immiscible in the polymer.

### 3.3. AFM

**Figure 4:**
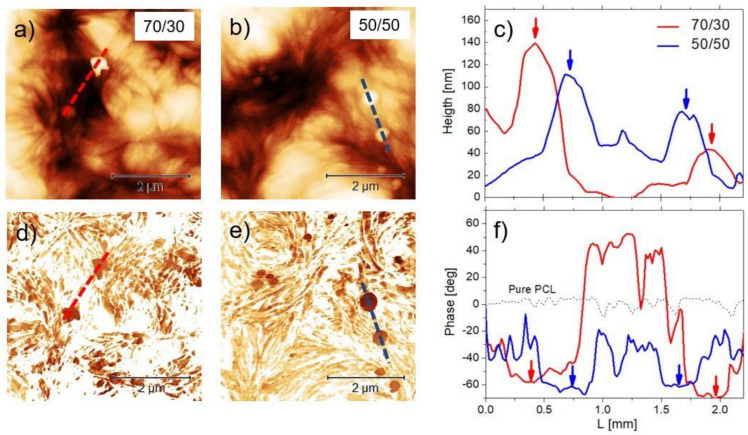
(**a**,**b**) 5 × 5 µm^2^ AFM height images of the 70/30 and 50/50 formulations with (**c**) cross-sectional traces taken on the two particles. (**d**,**e**) Phase images corresponding to the topographies above and (**f**) corresponding cross-sectional traces, including the signal corresponding to pure PCL. Arrows are a guide for the eye, indicating height maxima and corresponding phase minima in the two formulations.

**Figure 5:**
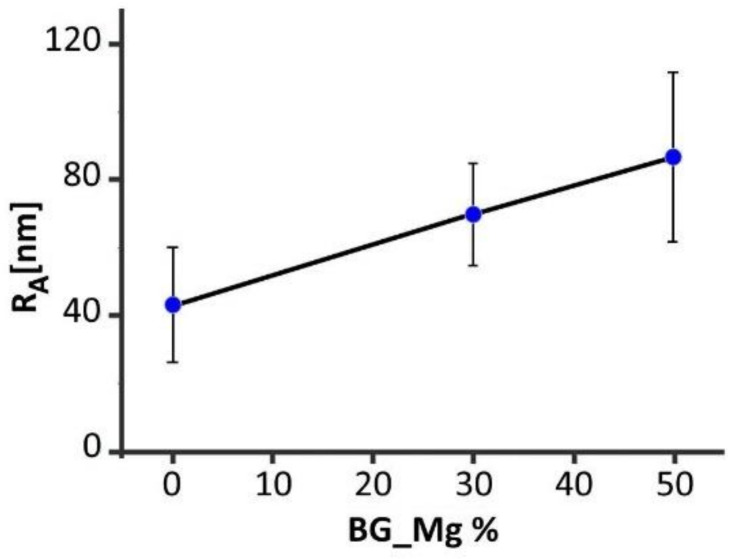
Roughness (R_A_) values in correspondence with the three formulations, i.e., pure PCL, 70/30, and 50/50.

AFM can be helpful to elucidate the small-scale aspect of the surface in relation to the presence and distribution of the BG-Mg particles, hence providing added values to the SEM findings. In Figure 4a,b, 5 × 5 µm^2^ topographic AFM images of both the 70/30 and 50/50 formulations are shown, respectively, whereas, in Figure 5, the R_A_ values corresponding to the three formulations are displayed. The topographies display well the intermixture between the fibre-like nature of the polymeric matrix and the BG particles; however, the latter are more clearly resolved in the phase images (Figure 4c,d) than in the height images. Cross-sectional traces of height and phase images in correspondence with the two particles are shown in Figure 4c,f, respectively; for comparison, a representative trace corresponding to the pure PCL phase signal was also reported [31].

The highest structural features in the height images of 70/30 were less than 120 nm, and the corresponding phase feature showed a negative phase shift of about 30° (red lines). The magnitude of the height variations in the 50/50 traces was within 60 and 40 nm, and the magnitude of the phase shift was within a negative variation of about 30° as well (blue lines). From Figure 5, one notes that R_A_ increased in correspondence with the increased particle fraction with respect to pure PCL; this agrees well with the visual impression given by the SEM images in Figure 2.

### 3.4. Nanomechanical Analysis

**Figure 6:**
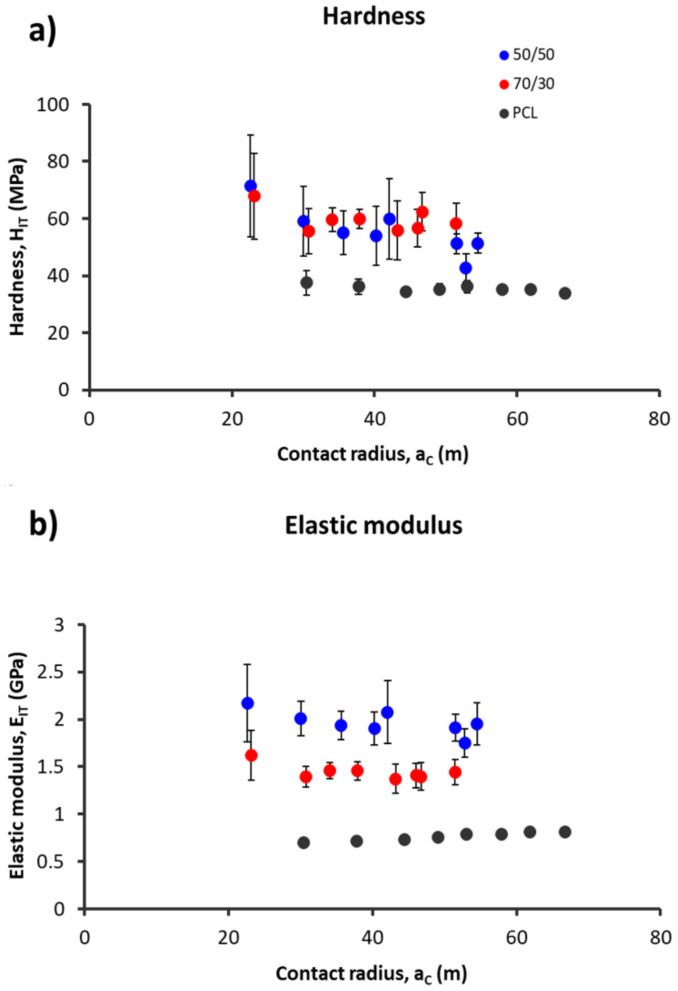
(**a**) Hardness (H) and (**b**) elastic modulus (E) data as a function of contact radius (a_C_) for the monolayer composites of 70/30 (blue dots) and 50/50 (red dots) and for the PCL control (black dots). Mean ± SD, n = 6.

Aiming to minimize the time-dependent effects of the polymer viscoelastic behaviour on the mechanical parameters estimation—assessed by fitting the experimental curves with the Dorner–Nix method [26]—preliminary indentations were carried out at increasing creep times (pause), i.e., from 25 to 600 s. These time-dependent effects were negligible for creep time values of 200 s for PCL and 70/30, whereas a time of 150 s was set for 50/50. E and H were evaluated considering various maximum loads (P_MAX_), ranging from 50 to 450 mN, i.e., with various contact radii (a_C_) (Figure 6).

The PCL fibres had a hardness (H = 35 ± 2 MPa) and an elastic modulus (E = 0.80 ± 0.05 GPa) lower than the composite materials’. The mechanical properties were obtained considering a contact radius (a_C_) in the range of 20—50 µm, hence the range was larger than the average grain size determined by SEM and AFM analyses. As concerns the 70/30 composite, the mechanical properties remained constant for a_C_ > 30 µm^2^ (H = 58 ± 8 MPa and E = 1.4 ± 0.2 GPa), whereas for the 50/50 composite, its values continued to decrease for a_C_ > 50 µm^2^ (H = 52 ± 11 MPa and E = 2.0 ± 0.2 GPa). Nevertheless, E mean values of the 50/50 composite were always greater than the 70/30 composite, whereas the H values were similar.

### 3.5. In Vitro Bioactivity

**Figure 7:**
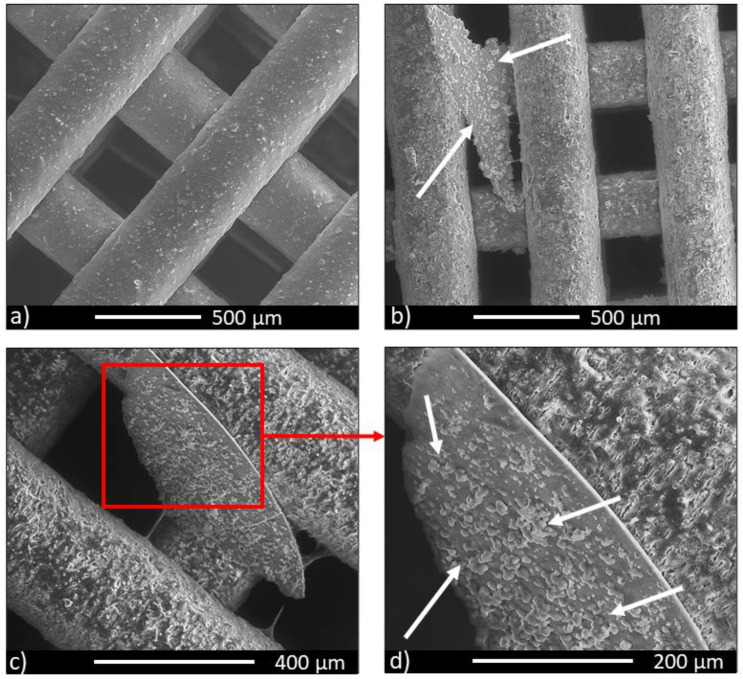
SEM images of the 50/50 scaffold: (**a**) non-immersed, (**b**) after soaking in SBF for 7 days, and (**c**,**d**) after soaking in SBF for 14 days. Arrows indicate HA precipitates.

The results of in vitro bioactivity in SBF of 5 × 5 × 3 mm^3^ composite scaffolds are reported in Figure 7 for increasing times.

Concerning the 70/30 sample composition, for up to 3 days there were no significant variations compared to the non-immersed samples; at 7 days, locally, a very thin layer of calcium phosphate mixed with other salts formed, but, according to the protocol developed by Kokubo and Takadama [30], this was not enough to confirm bioactivity. Focusing on the 50/50 samples, in vitro bioactivity was improved. After 7 (Figure 7b) and 14 (Figure 7c) days of immersion, the formation of sheets of carbonated hydroxyapatite took place. On the surface of the sheets the formation of globular precipitates was observed, with the typical morphology of hydroxyapatite in addition to clusters of hydroxyapatite (Figure 7d). EDS analysis confirmed the nature of the precipitates (in terms of the Ca/P ratio). According to Kokubo and Takadama’s protocol, these scaffolds can be classified as moderately bioactive. The results of the in vitro bioactivity were not carried out on the control group because of the bioinert nature of PCL [32].

### 3.6. Cell Viability Assay

**Figure 8:**
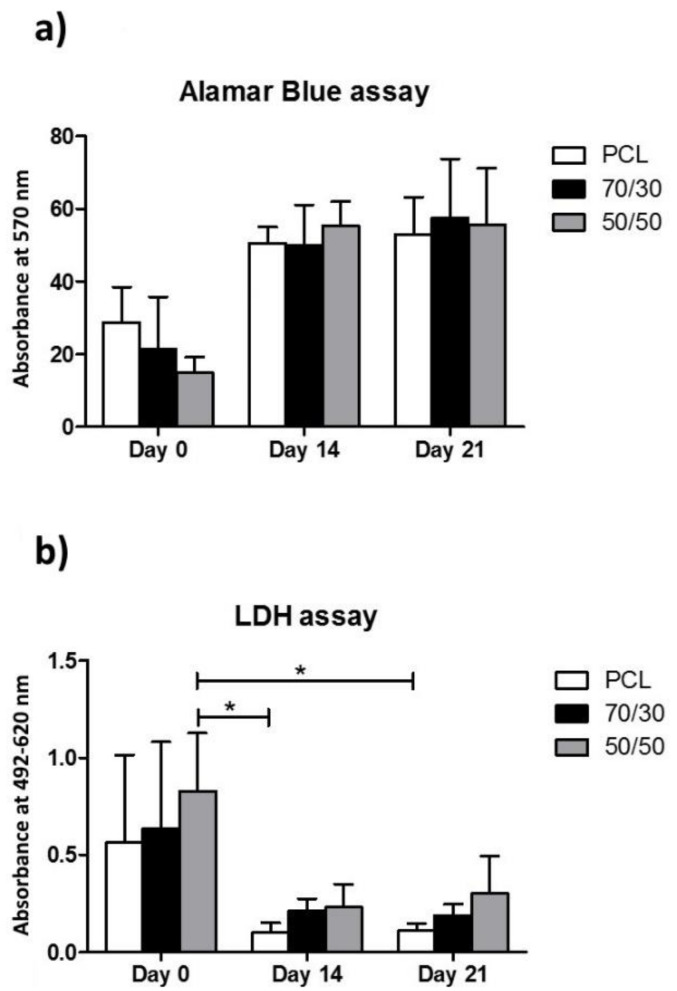
(**a**) Alamar Blue^®^ and (**b**) LDH assay to evaluate the cell vitality and cytotoxicity, respectively, of pure PCL, 70/30, and 50/50 scaffolds at 0, 14. and 21 days. Alamar Blue and LDH graphs were expressed as mean ± SD. Data were compared by the Kruskal–Wallis test, followed by Dunn’s post-hoc test, with * *p* < 0.05.

Data on the viability of the BM-MSCs seeded on the printed scaffold performed by Alamar Blue^®^ assay (Figure 8a) showed an increase in growth and metabolism of cells up to 21 days. A low cell viability was observed at day 0 for all the investigated materials. This evidence could be related to the hydrophobic characteristic of the PCL surface [33], which partially inhibits the cell adhesion. Nevertheless, at 21 days, cells were able to grow and to colonize the biomaterial, as shown by the increase in their viability (Figure 8).

### 3.7. Cytotoxic Assay

The cytotoxicity of the PCL, 70/30, and 50/50 scaffolds evaluated on the BM-MSCs through LDH assay is shown in Figure 8b. The differences in LDH levels were quite small for all printed materials at all experimental times. In particular, at day 0 the cytotoxicity was about 0.55, 0.63, and 0.82 for the PCL, 70/30, and 50/50 scaffolds, respectively; after 21 days, these values decreased significantly for all the materials (PCL ~0.10, 70/30 ~0.19, and 50/50 ~0.30). To allow cell adhesion onto hydrophobic scaffolds, the BM-MSCs were incubated in a small volume of the medium for 30 min at room temperature. This seeding method may have contributed to the higher lactate dehydrogenase release at day 0 with respect to the other times evaluated.

## 4. Discussion

The incorporation of bioactive glasses into PCL provided a class of hybrid biomaterials with remarkably improved mechanical properties, controllable degradation rates, and enhanced bioactivity, which are suitable for bone tissue engineering. This work presented a comprehensive and multifactorial characterization of a 3D-printed composite made of PCL and a novel Mg-doped BG for trabecular bone regeneration applications. The optimal printing parameter values are those that allow the obtainment of pore and fibre sizes closest to the theoretical ones, which were selected from the design established in our previous study [19] (Table 1). The performed microscopy analyses showed, in correspondence with the selected parameters, for the 70/30 formulation, an average PORE of 292 ± 12 µm and a FIBRE of 333 ± 18 µm, whereas, for the 50/50 formulation, an average PORE of 293 ± 14 µm and a FIBRE of 331 ± 14 µm were shown. According to our results, bearing in mind the perspective of manufacturing and testing entire 3D scaffolds for bone tissue engineering applications, the 50/50 composition should be preferred compared to the 70/30 composition, as it showed higher in vitro bioactivity, higher roughness, and a higher fibre elastic modulus compared to the 70/30 composition, even if the latter exhibited a slight improvement in the cellular viability. Formulations containing a higher percentage of BG-Mg than 50% were not considered because of the deriving limitations in the procedures both for the fabrication of a homogeneously dispersed bulk composite material and its processing through the extrusion technology, given the increased material viscosity and risk of clogging phenomena. Moreover, previous literature evidence [34] suggests that higher loading percentages (60% or higher) can lead to a decrease in the scaffold’s mechanical properties (i.e., toughness) because of the brittle behaviour of pure BG. On the other side, concentrations of BG particles that are not too low are to be preferred in order to improve the biological performance of the scaffold and to promote osteogenesis compared to composites with a small BG content (20% or less) [34].

Nanoindentation findings suggest that incorporation of bioactive glasses into PCL remarkably improves the mechanical properties of the composite. Comparison between the two composite formulations showed a greater value of the elastic modulus in the 50/50 composition, whereas the hardness values were not significantly different for both compositions (Figure 6). The elastic modulus describes the resistance to elastic deformation, whereas hardness is related to the plastic deformation. The increase in the amount of bioactive glass powder, which is considered a stiff and brittle material, within the polymer phase (ductile) causes a strong stiffening of the fibre without changing its plasticity. In this regard, two possible phenomena could be hypothesized: (i) the brittleness of the BG did not contribute to the increase in hardness between the two formulations [34] and (ii) the poor miscibility between the hydrophobic polymer and hydrophilic glass could generate an interfacial debonding [35]. Considering the fibre’s mechanical properties, the elastic modulus affects both the nano/micro- and the macro-scale behaviour of the scaffold. Regarding the nano/micro-scale, the elastic modulus strongly regulates adhesion, proliferation, apoptosis, and differentiation of cells; in particular, stronger adhesion, higher proliferation, and lower apoptosis rates are highlighted by cells in the presence of stiffer substrates compared to softer ones [36,37]. Moreover, the osteogenic differentiation of mesenchymal stem cells is enhanced on substrates with a higher elastic modulus [38]. Nevertheless, the reported studies focused on designing hydrogels—with an elastic modulus from tens of kPa to a few MPa—able to mimic the extracellular matrix for bone cell cultures, whereas the present study focused on composite scaffolds—with a higher elastic modulus (about 1.4 GPa for the 70/30 composition and 2.0 GPa for the 50/50 composition)—aiming to mimic the whole bone structure. Khatiwala et al. [37] observed that osteoblastic cells cultured on polystyrene proliferated twice as fast as those on hydrogel substrates, but this does not allow differentiation between the two polymeric substrates. Tan and Teoh [39] investigated the effect of PCL membrane stiffness on fibroblastic cell proliferation. Membrane stiffness ranged from 0.05 to 0.55 N/mm, and the fibroblasts preferred to proliferate in lower stiffness membranes (0.05–0.12 N/mm). However, also in this case, the stiffness values of the PCL membranes were too far from those calculated here (170 N/mm for the 70/30 and 220 N/mm for the 50/50, computed from the elastic modulus). At the macro-scale, the elastic modulus of the fibre is linearly related to the compressive modulus of the entire construct [19]. As a consequence, the compressive modulus of the 50/50 scaffold should be 0.100 GPa, whereas the 70/30 modulus should be 0.074 GPa. Scaffolds developed for trabecular bone applications require a compressive modulus in the range of 0.1–5 GPa [22]; accordingly, the 50/50 composition should be preferred to the 70/30 from this point of view.

Besides stiffness of the substrate, nano/micro-topography is also crucial, considering osteoblasts’ adhesion and mesenchymal stem cells’ differentiation [40]. Cells respond to a specific substrate topography depending on their size [41,42]. Although, because of the high aspect ratio of the surface, it is not trivial to determine the roughness on a scale of the order of the osteoblasts’ size (20–50 µm) or higher; it is reasonable that at these scales R_A_ would increase by about an order of magnitude with respect to the values shown in Figure 5 because of the strong inhomogeneity of the surface. This scenario is compatible with the observations of Faia-Torres et al. who focused on the role played by morphological features in osteoblasts’ attachment and differentiation on pure PCL [43]. In our situation, the addition of BG-Mg meets the requirements of increasing the overall ruggedness of the surface, which seems to create more favourable conditions for cell proliferation with respect to a smoother surface [44]. Yet, as confirmed by the AFM measurements, the small-scale’s contribution to such an increase was given by the presence of the particles, which, on average, contributed to an increase in roughness, as reported in Figure 5. The particles appeared densely and homogeneously distributed on the surface, with an average distance between the particles of about one or a few micrometers (Figure 4a,b), which was smaller than the osteoblasts’ size.

The ability of a material to form a CaP superficial layer when immersed in SBF is taken as an indication of its bioactivity [30]; moreover, this layer is able to support new bone growth at the interface between the bone and the implant. In this perspective, the in vitro bioactivity shown by the 50/50 composition could provide a greater potential of osseointegration [45,46,47].

In the present study, the incorporation of BG-Mg powder into the PCL matrix did not cause negative effects on cytotoxicity or inhibition of cell growth until day 21 (Figure 7 and Figure 8), considering both the investigated formulations. These results agreed with the cellular viability assays of the basic materials, i.e., PCL pellets (MW = 80,000, Aldrich Chemistry) and bioactive glass powder BG-Mg with the composition (in mol%): 47.2% SiO_2_, 2.6% P_2_O_5_, 2.3% Na_2_O, 2.3% K_2_O, 35.6% CaO, and 10% MgO [15,48]. Referring to the bioactive glass, it exerted a strong stimuli effect on cell growth and metabolism; in particular, magnesium ion (Mg^2+^) favours different biological processes including the regulation of active calcium transport [17]. Different studies demonstrated the key role of Mg^2+^ in bone remodelling and skeletal tissue development, firstly due to its involvement in the calcification process [16]. In the present study, BG-Mg was chosen with the aim to improve, in future applications, the bone mineral density at the implant-tissue interface. The slight improvement in cellular response for composites could be caused by a low amount of bioactive glass particles piercing and emerging from the polymer matrix (Figure 2n,o). However, the bioactive glass powder loaded into the PCL matrix stiffened and increased the surface roughness of the fibre.

The data discussed above allow us to conclude that the 50/50 composition could be a promising biomaterial for the production of scaffolds addressing trabecular bone engineering applications. The use of conventional approaches (such as solvent casting and particulate leaching, phase separation, electrospinning, freeze drying, etc.) in realizing scaffolds strongly affects the control of both the internal and external architecture of scaffolds, including pore size, pore morphology, and overall structure porosity [22]. Accordingly, PED was used here because of the benefits offered over conventional methods, such as high flexibility in shape and size, high reproducibility, capabilities of precise control over internal architecture down to the microscale level, and a customized design that can be tailored to specific patient needs [4]. Moreover, the use of an auger-screw-based extrusion technology provided specific advantages in processing the composite formulations, mitigating the aforementioned limitations related to high material viscosity as well as possible clogging phenomena deriving from high BG-Mg microparticles concentration. In conclusion, the optimization of the scaffold structure was previously investigated in terms of architecture through the combination of the Taguchi method and CAD drawing [19], and here it was investigated by varying the composition of the composite material.

Future work should address in vitro and in vivo performance of the realized constructs. For instance, the protocol developed by Kokubo and Takadama [30] is a tool widely used today for preliminary examining the bioactivity of new materials [49], but some criticisms have been expressed [50]; in particular, the SBF tests look too simplistic to simulate the complexity of a dynamic biological environment, and they may lead to false negative and false positive results. A more structured evaluation of cellular response on printed scaffold architectures shall be tested, with a combination of degradation, release, and mechanical fatigue assessments, which approach the physiological scenario. For instance, the release of ions, such as Ca^2+^; PO_3_^2−^, Mg^2+^, and Si^+^, is interesting considering osteogenesis and angiogenesis, as reported in Rahaman, et al. [51] and Bellucci, et al. [48]. Finally, the in vivo behaviour should be evaluated in preclinical tests. Moreover, a future optimization should include the analysis of potential residual stresses because of the mismatch of coefficients of thermal expansion (CTE) of the matrix and of the glass phase in the composites [52,53], and eventually the glass composition should be tailored to this aim.

## 5. Conclusions

In this work, 3D-PCL-based composite scaffolds, containing a magnesium-doped bioactive glass, were produced and fully characterised. Such scaffolds were obtained thanks to an additive manufacturing technique, namely, precision extrusion deposition, which was able to print structures with precise replication of bone features in a repeatable and automatic fashion. Two different compositions (i.e., 70/30 and 50/50 PCL/BG) were thoroughly analysed with the aim of finding the optimal one with reference to morphological, mechanical, and biological performance.

Mechanical testing via nanoindentation pointed out that the incorporation of bioactive glass particles into PCL remarkably improved the mechanical properties of the samples. In particular, the 50/50 composition displayed a compressive modulus in the range of 0.1–5 GPa, as required for trabecular bone applications. Thus, such scaffolds are able to support mechanical compressive loads in the same range as trabecular bone tissue. Additionally, from the point of view of nano/micro-topography-crucial features for osteoblasts’ adhesion and colonization and mesenchymal stem cells’ differentiation versus bone lineage, the 50/50 composition produced results superior to the 70/30 composition.

SBF tests, which are often used as indicators of bioactivity, revealed that the 50/50 composition could offer a greater potential for osseointegration compared to the 70/30 composition. Moreover, biological tests conducted with human bone marrow mesenchymal stem cells assessed that the incorporation of BG particles into the PCL matrix did not cause any negative effect on cell viability or inhibition of cell growth, both the composite compositions being fully biocompatible and non-cytotoxic.

In conclusion, on the basis of the obtained results, the composite, printed scaffolds with 50/50 composition represent a promising alternative to realise patient-specific bone defects with tailored properties, in particular for the production of manufacts addressing trabecular bone engineering applications.

## Figures and Tables

**Table 1:** Scaffold printing parameters for 70/30, 50/50, and pure PCL composites.

Composite Material	Nozzle Diameter (µm)	Barrel Temperature (°C)	Printing Speed (rpm)	Extrusion Rate (rpm)
70/30	330	105	1	20
50/50	330	115	1	17
PCL	330	115	4	18

## Data Availability

Not applicable.
